# Factors Associated with D-Dimer Levels in HIV-Infected Individuals

**DOI:** 10.1371/journal.pone.0090978

**Published:** 2014-03-13

**Authors:** Álvaro H. Borges, Jemma L. O’Connor, Andrew N. Phillips, Jason V. Baker, Michael J. Vjecha, Marcelo H. Losso, Hartwig Klinker, Gustavo Lopardo, Ian Williams, Jens D. Lundgren, for the INSIGHT SMART and ESPRIT Study Groups and the SILCAAT Scientific Committee

**Affiliations:** 1 Centre for Health & Infectious Diseases Research (CHIP), Department of Infectious Diseases, Rigshospitalet, University of Copenhagen, Copenhagen, Denmark; 2 Research Department of Infection and Population Health, University College London, London, United Kingdom; 3 Hennepin County Medical Center, Minneapolis, Minnesota, United States of America; 4 Department of Medicine, University of Minnesota, Minneapolis, Minnesota, United States of America; 5 Veterans Affairs Medical Center, Washington, D. C., United States of America; 6 Hospital JM Ramos Mejia, Buenos Aires, Argentina; 7 University of Würzburg Medical Center, Würzburg, Germany; 8 Fundación Centro de Estudios Infectológicos, Buenos Aires, Argentina; 9 Centre for Sexual Health & HIV Research, University College London, London, United Kingdom; University of Pittsburgh Center for Vaccine Research, United States of America

## Abstract

**Background:**

Higher plasma D-dimer levels are strong predictors of mortality in HIV+ individuals. The factors associated with D-dimer levels during HIV infection, however, remain poorly understood.

**Methods:**

In this cross-sectional study, participants in three randomized controlled trials with measured D-dimer levels were included (N = 9,848). Factors associated with D-dimer were identified by linear regression. Covariates investigated were: age, gender, race, body mass index, nadir and baseline CD4^+^ count, plasma HIV RNA levels, markers of inflammation (C-reactive protein [CRP], interleukin-6 [IL-6]), antiretroviral therapy (ART) use, ART regimens, co-morbidities (hepatitis B/C, diabetes mellitus, prior cardiovascular disease), smoking, renal function (estimated glomerular filtration rate [eGFR] and cystatin C) and cholesterol.

**Results:**

Women from all age groups had higher D-dimer levels than men, though a steeper increase of D-dimer with age occurred in men. Hepatitis B/C co-infection was the only co-morbidity associated with higher D-dimer levels. In this subgroup, the degree of hepatic fibrosis, as demonstrated by higher hyaluronic acid levels, but not viral load of hepatitis viruses, was positively correlated with D-dimer. Other factors independently associated with higher D-dimer levels were black race, higher plasma HIV RNA levels, being off ART at baseline, and increased levels of CRP, IL-6 and cystatin C. In contrast, higher baseline CD4^+^ counts and higher high-density lipoprotein cholesterol were negatively correlated with D-dimer levels.

**Conclusions:**

D-dimer levels increase with age in HIV+ men, but are already elevated in women at an early age due to reasons other than a higher burden of concomitant diseases. In hepatitis B/C co-infected individuals, hepatic fibrosis, but not hepatitis viral load, was associated with higher D-dimer levels.

## Introduction

Chronic inflammation and activated coagulation are well-known features of HIV infection [1], [2] and evidence has accrued indicating that both processes contribute to an increased risk of death. Out of a panel of inflammatory and coagulation biomarkers tested in participants of the Strategies for Management of Antiretroviral Therapy (SMART) study [3], D-dimer, a fibrin degradation product, was the most predictive biomarker of overall mortality [4]. Furthermore, elevated D-dimer levels were found to be strongly associated with early mortality following ART initiation among severely immunosuppressed South-African patients [5].

A strong association between HIV replication and raised D-dimer levels has been demonstrated. D-dimer levels decline following antiretroviral therapy (ART) initiation [1], [5], [6] and increase after stopping ART in treatment experienced patients [1], [4]. Correlations of D-dimer with HIV viremia and markers of endothelial dysfunction and microbial translocation [1], [4], [7], [8] have also been reported. This favors the hypothesis that HIV replication and microbial translocation are among the main determinants of the hypercoagulable state seen in HIV-infected persons.

On the other hand, correlations with other biomarkers may also indicate that elevations of D-dimer levels are not mainly determined by HIV infection, but just reflect the presence of co-morbidities or unmeasured confounders that are truly associated with activated coagulation. Indeed, an increase of D-dimer levels with age has been reported in both HIV+ and HIV- individuals [9], [10] and it has been hypothesized that a higher burden of co-morbidities and an age-related pro-inflammatory state could explain this [11], [12]. Given the complex interaction of multiple factors leading to inflammation, endothelial dysfunction and activated coagulation in persons aging with HIV [13], questions remain as to what is the individual contribution of HIV-specific factors, demographics, co-infections and co-morbidities to the variance in D-dimer levels.

The purpose of this study is to identify factors independently associated with D-dimer levels in a large group of HIV+ individuals. Our main *a priori* hypotheses were that (1) the higher levels of D-dimer seen in older individuals are mainly attributable to a higher burden of co-morbidities and enhanced inflammation, and (2) that HIV-specific variables (HIV viremia, CD4^+^ cell count and ART use) are independently associated with higher D-dimer levels and that this association remains strong after adjustment for demographics, co-morbidities, smoking, and biomarkers of inflammation and renal function.

## Materials and Methods

The present study used baseline data from participants in three randomized controlled trials: (1) SMART (ClinicalTrials.gov number, NCT00027352) [3]; (2) Evaluation of Subcutaneous Proleukin in a Randomized International Trial (ESPRIT) (ClinicalTrials.gov number, NCT00004978); and (3) Subcutaneous Recombinant, Human Interleukin-2 in HIV-Infected Patients with Low CD4^+^ Counts under Active Antiretroviral Therapy (SILCAAT) (ClinicalTrials.gov number, NCT00013611) [14], whose methods have been described in detail elsewhere. Briefly, the SMART trial compared, in 5,472 individuals with CD4^+^ >350 cells/mm^3^ at baseline, continuous use of ART with structured treatment interruption guided by CD4^+^ cell count, involving stopping ART when CD4^+^ was >350 cells/mm^3^ and re-initiating ART when CD4^+^ was <250 cells/mm^3^ **.** The ESPRIT and SILCAAT trials compared IL-2 plus ART with ART alone in 4,111 individuals with CD4^+^ >300 cells/mm^3^ and 1,695 individuals with CD4^+^ between 50 and 299 cells/mm^3^, respectively. Participants from all three trials who had consented to storing blood for future research and whose serum D-dimer levels were measured at baseline (N = 9,848) were included in this study.

The SMART, ESPRIT and SILCAAT studies, including the consent for stored specimens, was approved by the institutional review board or ethics committee of each clinical site and of the University of Minnesota. A written informed consent was obtained from all participants involved in the three trials.

### Biomarker Measurements

In SMART participants, D-dimer, CRP, IL-6 and cystatin C were measured at the Laboratory for Clinical Biochemistry Research at the University of Vermont (Burlington). D-dimer levels were measured with immunoturbidometric methods on the Sta-R analyzer, Liatest D-DI (Diagnostic Stago, Parsippany, New Jersey, USA). IL-6 was measured with Chemiluminescent Sandwich ELISA (R&D Systems, Minneapolis, Minnesota, USA), CRP with a NBTMII nephelometer, N Antiserum to Human CRP (Siemens Diagnostics, Deerfield, Illinois, USA) and cystatin C with a BNII nephelometer (Dade Behring Inc., Deerfield, Illinois, USA). In the ESPRIT and SILCAAT trials, laboratory measurements were performed by SAIC-Frederick (Frederick, Maryland, USA). D-dimer was measured using an enzyme-linked fluorescent assay (ELISA) on a VIDAS instrument (bioMerieux Inc., Durham, North Carolina, USA), and CRP and IL-6 were measured using ELISA (R&D Systems, Minneapolis, Minnesota, USA). In SMART, lower limits of detection for IL-6, CRP, D-dimer and cystatin C were 0.16 pg/mL, 0.16 µg/mL, 0.01 µg/mL and 0.195 mg/dL, respectively. In ESPRIT and SILCAAT, lower limits of detection for IL-6, CRP and D-dimer were 0.156 pg/mL, 0.078 µg/mL and 0.045 µg/mL. The assays used to measure D-dimer and CRP, while different, compared very well on 20 duplicate samples. Estimated glomerular filtration rate was calculated using the Cockcroft-Gault formula [15] in ESPRIT and SMART participants. Total cholesterol, low-density lipoprotein cholesterol (LDLc) and high-density lipoprotein cholesterol (HDLc) were measured in SMART by Quest Diagnostics, Inc. (Madison, NJ) using standard enzymatic methods. LDLc was directly measured. Samples were not required to be fasting and were analyzed blinded to treatment arm.

The screening of SMART and ESPRIT participants for co-infection with hepatitis B (HBV) or hepatitis C (HCV) has been reported elsewhere [16]. Baseline plasma obtained from individuals with antibody tests positive for HBV and HCV was analyzed for levels of HCV RNA and HBV DNA using branched DNA assays (Versant HCV RNA 3.0 and Versant HBV DNA 3.0, respectively; Bayer Diagnostics), whose lower limits of detection were 615 and 357 IU/mL, respectively. Participants with a positive HBV/HCV antibody and/or viral load test were considered to have hepatitis co-infection. Baseline hyaluronic acid levels were measured in co-infected patients using an enzyme-linked binding protein assay (Corgenix, Colorado, USA) with a lower limit of detection of 10 ng/mL [17].

### Statistical Analyses

Factors independently associated with elevated D-dimer levels were identified by multivariable linear regression models. The distributions of D-dimer, CRP and IL-6 were right-skewed; thus a logarithmic transformation was used in the analyses. Log_2_-transformed D-dimer levels were modeled as the outcome. PROC REG was used in SAS (version 9.3; SAS Institute, Cary, NC, USA) to produce estimates with 95% confidence intervals (CI) to assess the contribution of covariates. Estimates were then exponentiated in order to correspond to fold differences in D-dimer levels per unit or category difference in the covariates included in the linear regression models. The impact of inter-study, inter-laboratory and inter-method variability was minimized by entering terms for each study in all models. The goodness of fit of the models was assessed using the adjusted R^2^ coefficient. A two-sided *P*-value of <0.05 was used as the threshold of statistical significance.

As data on some variables of interest were not collected in all three trials, the regression models were fitted to three different datasets:

1. The largest dataset combining SMART, ESPRIT and SILCAAT participants (N = 9,848) included: age, gender, race, body mass index (BMI), CD4^+^ cell counts (nadir and baseline), markers of inflammation (CRP and IL-6), ART use and ART regimens;
2. A smaller dataset consisting of SMART and ESPRIT participants (N = 6,928) included: co-morbidities (HBV and HCV, diabetes mellitus, prior cardiovascular disease; defined as prior myocardial infarction, stroke or coronary artery disease requiring surgical procedure) and renal function (eGFR);
3. The smallest dataset consisting only of SMART participants (N = 4,488), included: smoking, cholesterol levels (LDLc and HDLc) and additional information on renal function (cystatin C).

Given the significantly higher levels of D-dimer seen in women, we found it helpful to investigate if determinants of D-dimer levels could differ in analyses stratified by gender. Since the three trials involved participants with different baseline characteristics, we also investigated interactions between D-dimer levels, study (SMART, ESPRIT and SILCAAT), plasma HIV RNA levels, inflammatory biomarkers (IL-6 and CRP) and demographic covariates found to be correlated with D-dimer levels (age, race and gender).

In the subset of hepatitis co-infected individuals, we sought to investigate the contribution of liver fibrosis and replication of hepatitis viruses to the variance of D-dimer levels. We then entered hyaluronic acid levels (a validated marker of hepatic fibrosis, which was measured at baseline in 245 study participants co-infected with HBV and in 860 co-infected with HCV), as well as HBV and HCV viral load, into models adjusted for demographics (age, gender and race) and restricted to HBV- and HCV- co-infected participants, respectively.

### Sensitivity Analyses

Correlates of D-dimer levels were also investigated by using multivariable logistic regression models. Participants were dichotomized into two groups: low and elevated D-dimer levels; the latter defined as levels greater than 0.377 µg/mL (4^th^ quartile for the participants in all three trials). Odds ratio (OR) with 95% CI were calculated to assess the contribution of correlates.

We also carried out two additional sensitivity analyses using linear regression models: (a) stratified by current ART use (i.e., yes versus no) and (b) stratified by plasma HIV RNA levels (i.e., plasma HIV RNA ≤500 versus >500 copies/mL). Analysis (a) was performed in order to investigate the effect of plasma HIV RNA on D-dimer levels, since plasma HIV RNA was not included in the primary analyses because of the possibility that the colinearity between ART use and plasma HIV RNA levels could affect valid interpretation of our findings. Analysis (b) was performed to determine whether the suppression of viral replication would change the predictors of D-dimer levels.

## Results

Baseline demographic, clinical and laboratory characteristics are summarized separately for each of the three datasets included in the analyses and are presented in Table 1. In analyses investigating possible interactions between study and demographic covariates, the following interactions were found to be significant: study and gender (p = 0.0005), and study and race (p<.0001). There was, however, no evidence of an interaction between study and age (p = 0.20). Because the interactions suggested only moderate differences in effect and the biomarker assays compared well on duplicates, we found it appropriate to fit models to datasets pooling the three trials. Moreover, given that our main results are fairly consistent between datasets which used the two different D-dimer assays we believe that the associations presented in this study are not artificially influenced by the use of different assays.

**Table 1:** Baseline Characteristics by Dataset ESPRIT, SILCAAT and SMART Patients.

	SMART, ESPRIT &SILCAAT (N = 9,848)	SMART & ESPRIT(N = 6,928)	SMART(N = 4,488)
**D-dimer (median, IQR) (µg/mL)**	**0.24 (0.15–0.38)**	**0.22 (0.15–0.37)**	**0.20 (0.13–0.36)**
**Demographics**			
Age in Years (median, IQR)	42 (36–49)	42 (36–49)	44 (38–50)
Female Gender (%)	21.8	23.2	25.5
Black Race (%)	19.4	21.3	27.8
BMI (median, IQR)	24.34 (22.12–27.00)	24.45 (22.15–27.30)	24.99 (22.50–28.09)
**HIV-specific variables**			
Baseline CD4+ cell count (median, IQR) (cells/mm^3^)	490 (368–671)	540 (422–722)	601 (470–799)
Nadir CD4+ cell count (median, IQR) (cells/mm^3^)	200 (84–316)	229 (121–335)	250 (154–358)
Plasma HIV RNA ≤500 copies/mL (%)	76.4	76.3	73.3
ART regimen			
Off ART (%)	8.3	10.1	15.2
PI-based (%)	37.5	33.7	31.8
NNRTI-based (%)	36.5	38.9	37.8
Other (%)	17.7	17.3	15.2
**Biomarkers of Inflammation**			
IL-6 (median, IQR) (pg/mL)	1.80 (1.20–2.89)	1.81 (1.17–2.90)	1.72 (1.07–2.93)
CRP (median, IQR) (µg/mL)	1.59 (0.70–3.67)	1.61 (0.71–3.76)	1.70 (0.71–4.07)
**Co-morbidities**			
Cardiovascular disease (%)*	n/a	2.7	3.6
Diabetes Mellitus (%)*	n/a	5.3	6.7
Hepatitis B (%)*	n/a	3.7	2.2
Hyaluronic Acid (median, IQR) (ng/mL)***	n/a	23.80 (14.63–43.69)	n/a
HBV DNA (median, IQR) (IU/mL)***	n/a	71,704 (2,000–100,000,000)	n/a
Hepatitis C (%)*	n/a	14.4	13.4
Hyaluronic Acid (median, IQR) (ng/mL)****	n/a	33.17 (18.75–59.82)	n/a
HCV RNA (median, IQR) (IU/mL)****	n/a	2,576,804 (583,936–7,610,964)	n/a
Smoking**	n/a	n/a	40.5
**Renal Function**			
eGFR (median, IQR) (mL/min per 1.73 m^2^)*	n/a	111.56 (100.66–121.03)	110.82 (100.25–120.56)
Cystatin C (median, IQR) (mg/dL)**	n/a	n/a	0.81(0.71–0.92)
**Cholesterol Levels**			
Total Cholesterol (median,IQR) (mg/dL)**	n/a	n/a	192 (164–222)
LDL-c (median,IQR) (mg/dL)**	n/a	n/a	112 (90–137)
HDL-c (median,IQR) (mg/dL)**	n/a	n/a	40 (33–51)

*Not ascertained for patients in SILCAAT.

**Not ascertained for patients in SILCAAT or ESPRIT.

***Data available for n = 245 participants.

****Data available for n = 860 participants.

### Demographics

**Figure 1:**
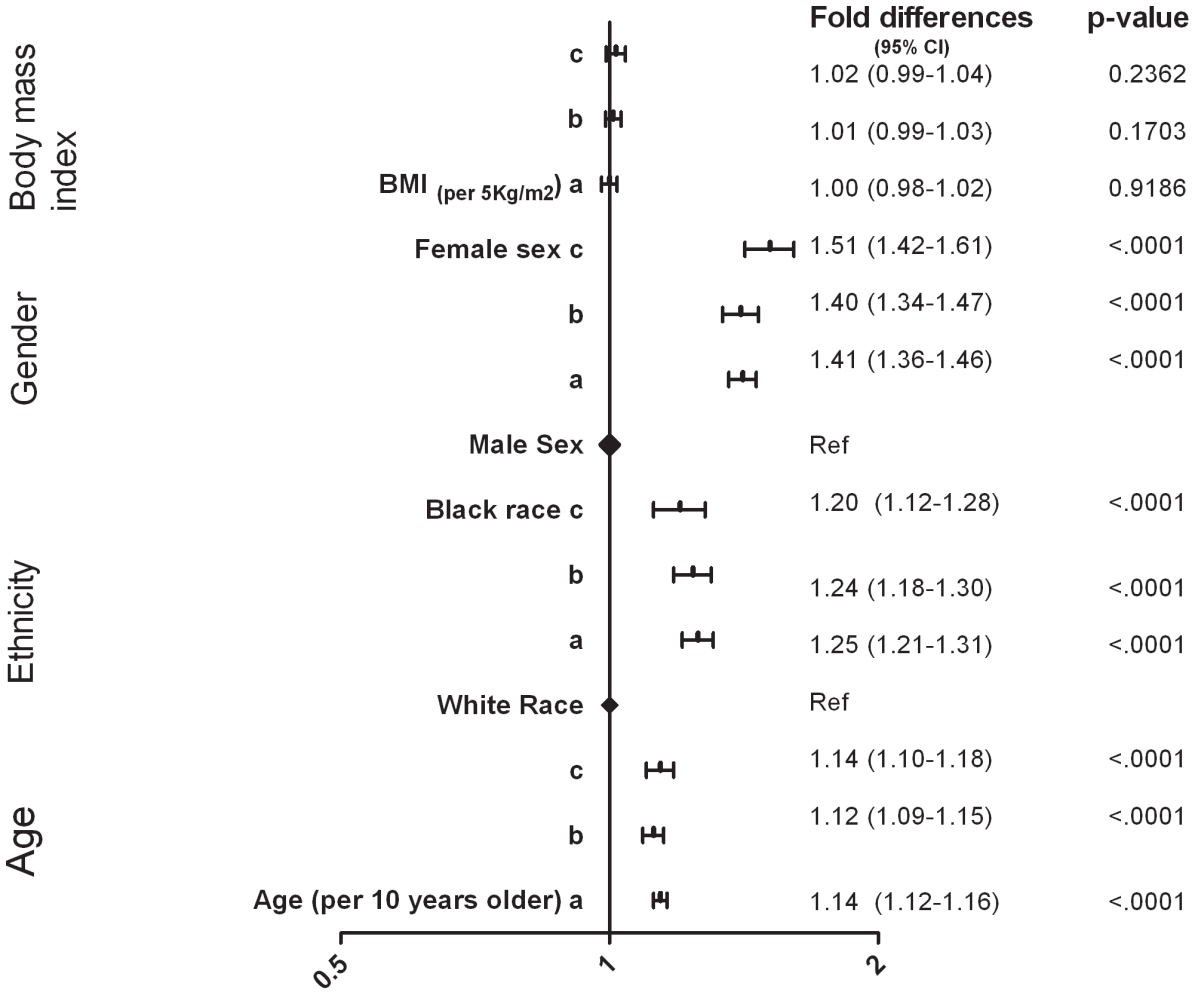
Demographics and D-dimer levels. (a) SMART/ESPRIT/SILCAAT; adjusted for demographics, HIV-specific variables and biomarkers of inflammation. (b) SMART/ESPRIT; as in (a) and also adjusted for co-morbidities (CVD, DM and hepatitis B/C) and eGFR. (c) SMART only; as in (b) and also adjusted for smoking, cystatin C and cholesterol levels.

The demographic factors found to be positively and independently correlated with D-dimer levels were older age, black race and female sex. The results were robust, with similar fold differences seen consistently across multiple models using different datasets and after adjustment for an increasing range of covariates (Figure 1). BMI, on the other hand, was not found to be associated with D-dimer levels.

**Figure 2:**
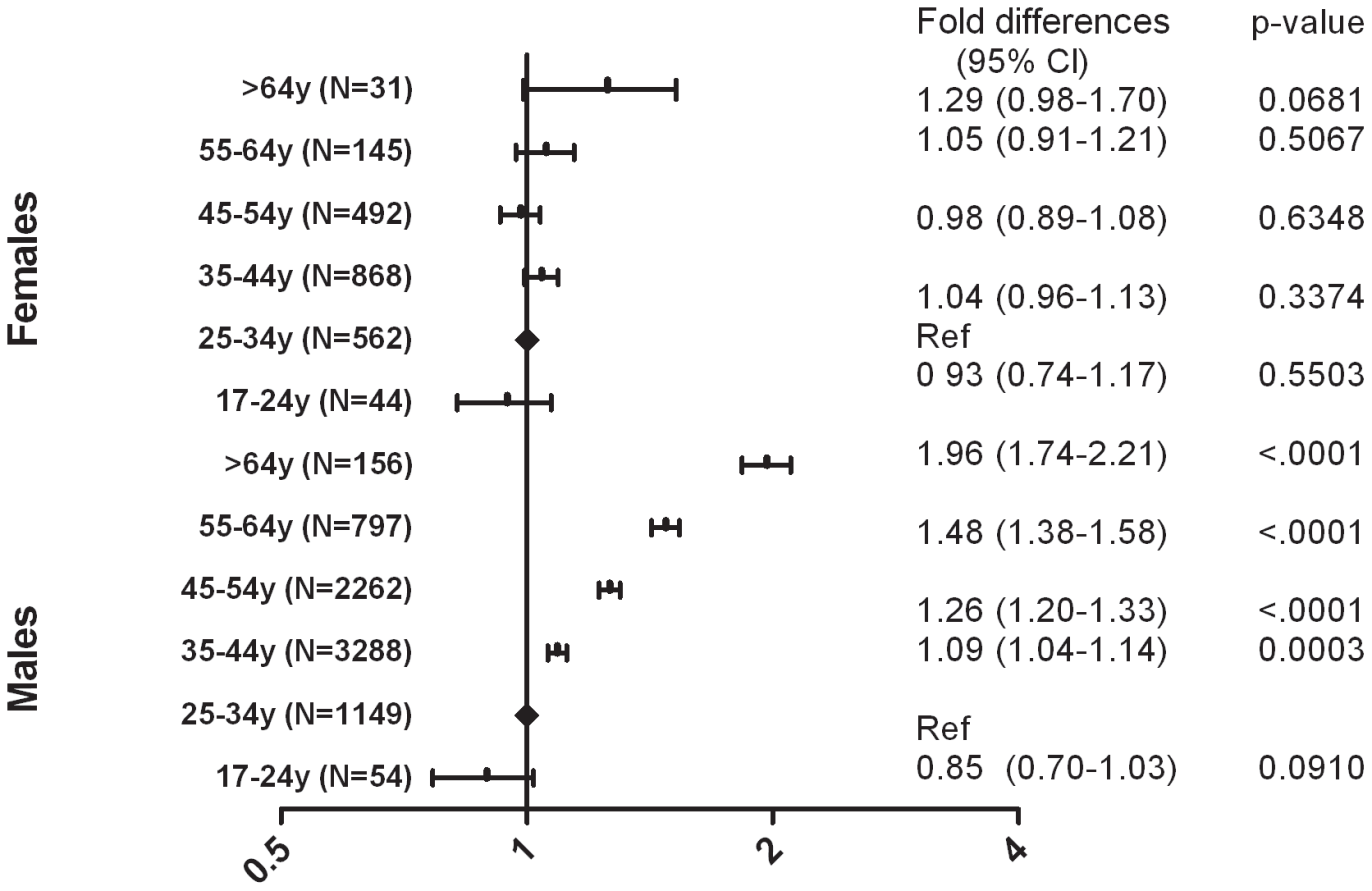
Figure 2. D-dimer levels across age groups stratified by gender (a). (a) SMART/ESPRIT/SILCAAT; adjusted for demographics, HIV-specific variables and biomarkers of inflammation.

In analyses stratified by gender, older age was found to be independently associated with higher D-dimer levels; the effect of increasing age on D-dimer was, however, much stronger in men than in women (Figure 2). Women in all age groups were found to have significantly higher D-dimer levels when compared to males aged 25–34 years (data not shown). The interaction between age and gender was found to be significant (p<.001), but there was no evidence of an interaction between age and plasma HIV RNA (p = 0.40) and between age and biomarkers of inflammation (p = 0.98 for IL-6 and p = 0.33 for CRP).

The addition of an increasing number of covariates in multiple regression models consisting of SMART and ESPRIT datasets did not substantially change the power to predict D-dimer levels (adjusted R^2^ values ranged from 0.15 to 0.22).

### HIV-specific Variables

**Figure 3:**
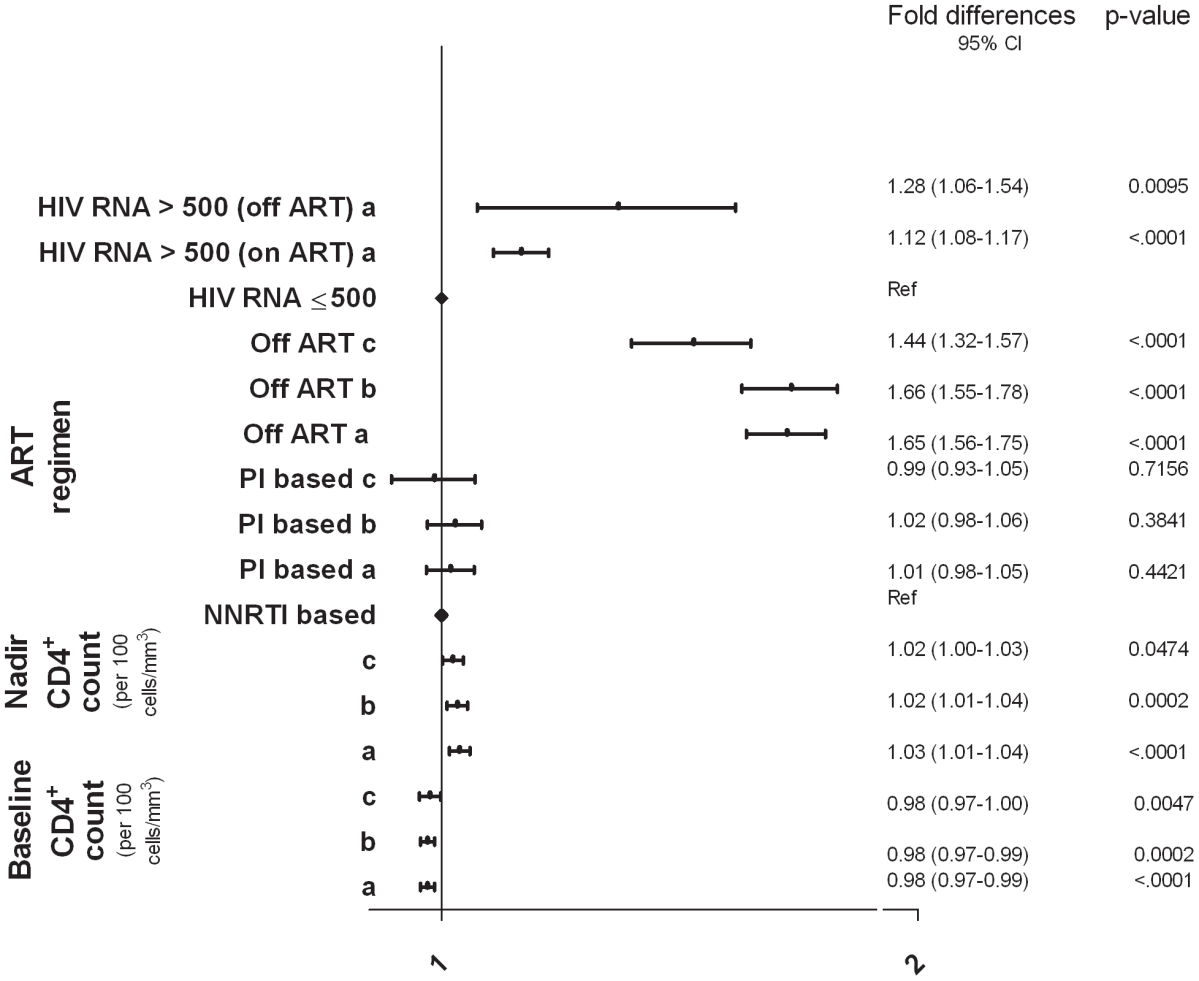
HIV-specific variables and D-dimer levels. (a) SMART/ESPRIT/SILCAAT (N = 9848; 821 of whom were off ART at baseline); adjusted for demographics, HIV-specific variables and biomarkers of inflammation. (b) SMART/ESPRIT (N = 6928); as in (a) and also adjusted for co-morbidities (CVD, DM and hepatitis B/C) and eGFR**.** (c) SMART (N = 4488); as in (b) and also adjusted for smoking, cystatin C and cholesterol levels.

Uncontrolled HIV infection, as demonstrated by lower baseline CD4^+^ cell counts and higher plasma HIV RNA, was found to be positively correlated with higher D-dimer levels. This could well explain why being off ART at baseline was also independently associated with elevated D-dimer. We also found a positive and independent correlation between nadir CD4^+^ cell counts and D-dimer levels. Among those on ART, protease inhibitor (PI)-based and non-nucleoside reverse transcriptase inhibitor (NNRTI)-based regimens were associated with similar D-dimer levels and no significant differences were noted (Figure 3).

### Biomarkers of Inflammation

**Figure 4:**
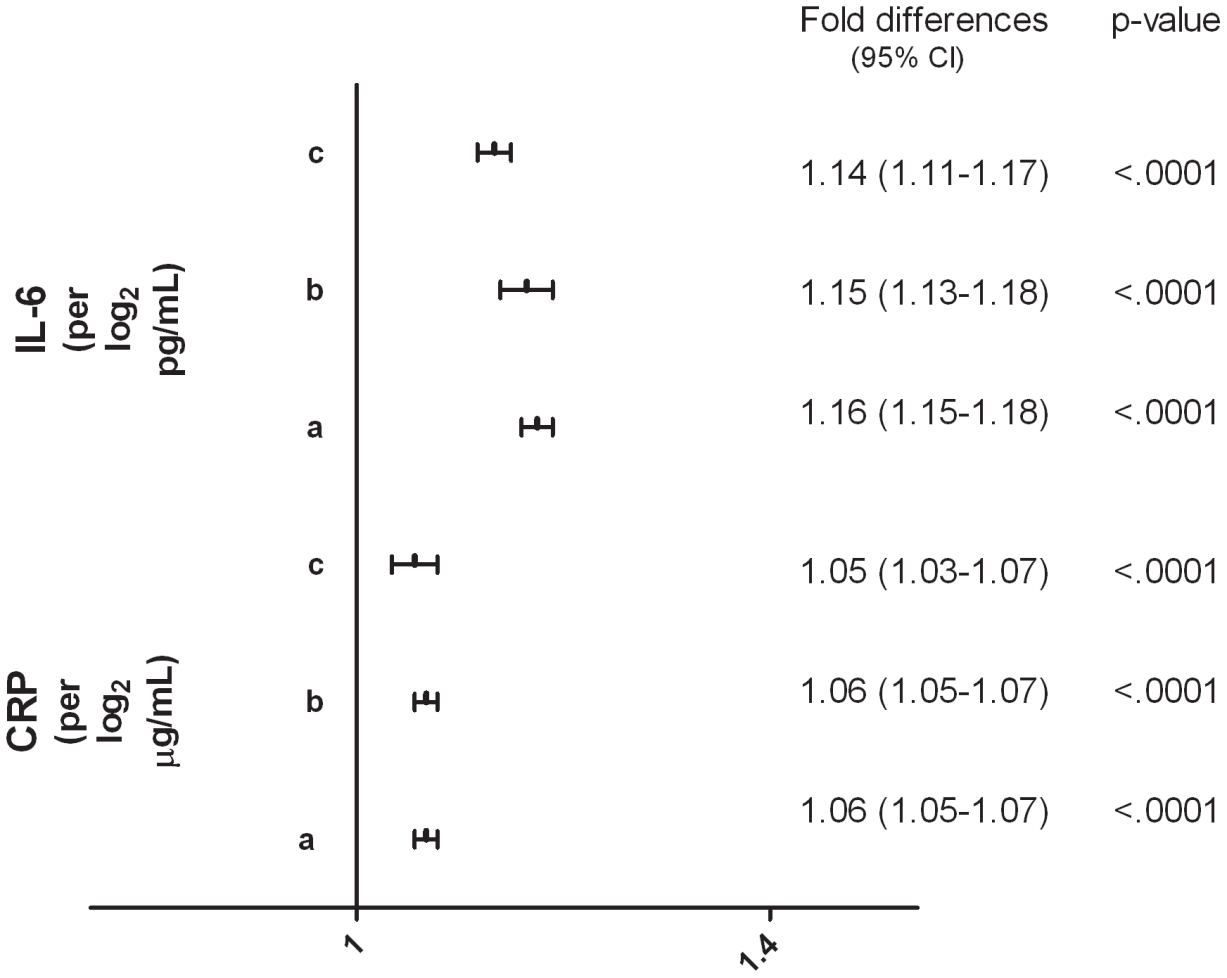
Biomarkers of Inflammation and D-dimer levels. (a) SMART/ESPRIT/SILCAAT (N = 9848); adjusted for demographics, HIV-specific variables and biomarkers of inflammation**.** (b) SMART/ESPRIT (N = 6928); as in (a) and also adjusted for co-morbidities (CVD, DM and hepatitis B/C) and eGFR. (c) SMART (N = 4488); as in (b) and also adjusted for smoking, cystatin C and cholesterol levels.

**Figure 5:**
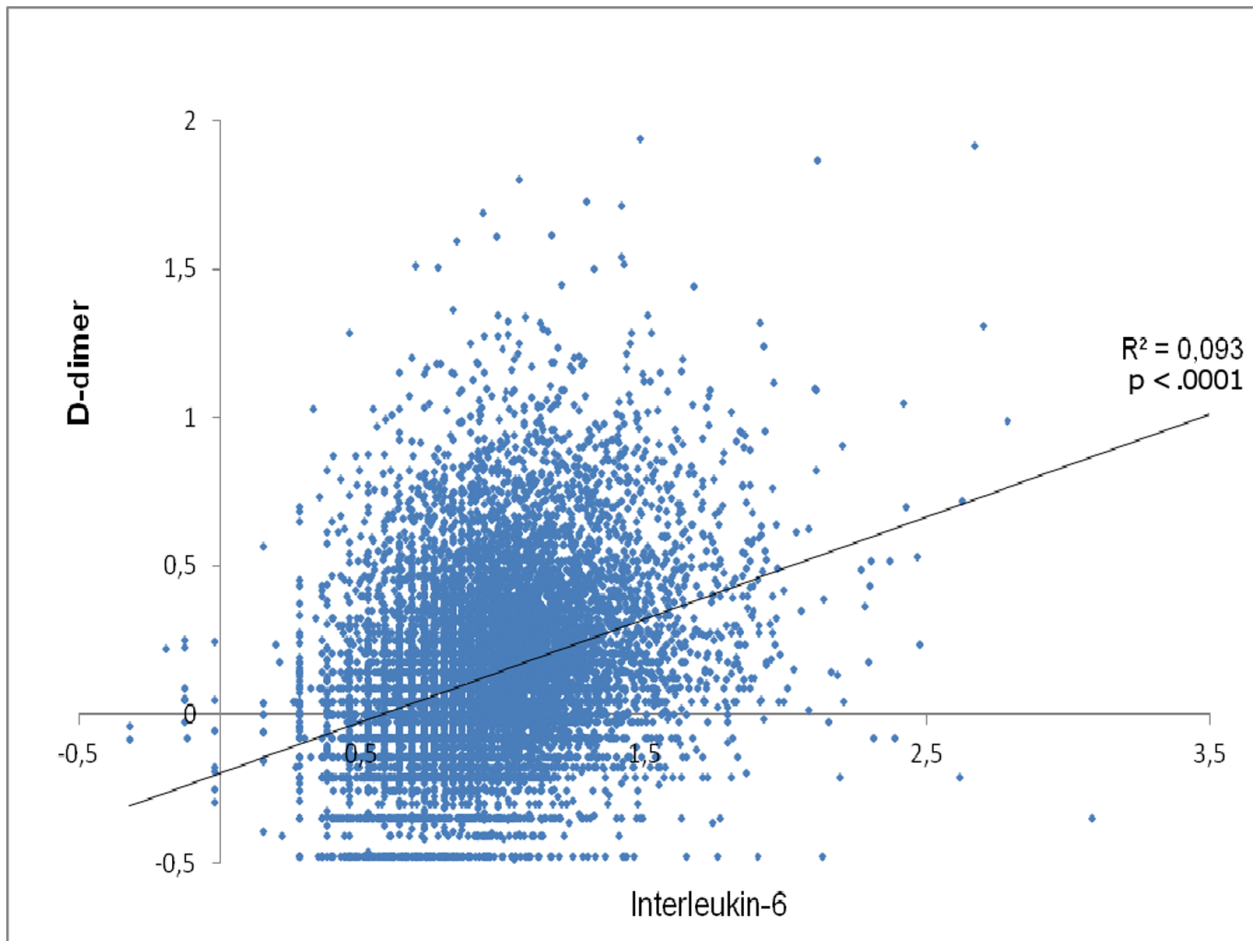
Correlation between D-dimer and IL-6 levels*. * Plotted values refer to log_10_ transformed levels of units of measurement based on the molecular masses of D-dimer and IL-6 (nmol/L for D-dimer and fmol/L for IL-6).

Both CRP and IL-6 were independently and positively correlated with D-dimer. Once again, the results were robust and observed consistently in all linear regression models (Figure 4). The linear positive relationship between IL-6 and D-dimer levels is graphically illustrated in Figure 5.

### Co-morbidities, Renal Function and Cholesterol Levels

Prior cardiovascular disease (Fold Difference 0.90, 95% CI [0.79–1.03], p = 0.14), smoking (0.98, [0.93–1.03], p = 0.45) and diabetes mellitus (0.94, [0.85–1.04], p = 0.23) did not have a significant association with D-dimer levels in adjusted models. On the other hand, HBV (1.27, [1.07–1.50], p = 0.0061) and HCV (1.19, [1.10–1.29], p<.0001) co-infection were independently associated with raised D-dimer levels. In co-infected participants, the degree of liver damage, as demonstrated by higher hyaluronic acid levels (1.05[1.01–1.09] per 1 log_2_ ng/mL, p = 0.0078, for HBV and 1.05[1.00–1.09], p = 0.0315, for HCV), but not the viral load of hepatitis viruses (1.01[1.00–1.02] per 1 log_2_ IU/mL, p = 0.17, for HBV and 0.99[0.97–1.00], p = 0.09, for HCV) was found to be positively correlated with D-dimer levels.

Higher eGFR levels at baseline were found to be significantly associated with lower D-dimer in the dataset consisting of SMART and ESPRIT participants (0.99 [0.98–1.00] per 10 mL/min per 1.73 m^2^, p = 0.023). However, in SMART participants, after further adjustment for cystatin C, as well as for smoking and cholesterol levels, the association between higher eGFR count and lower D-dimer was no longer significant (1.02, [1.00–1.04], p = 0.06). Higher cystatin C levels were strongly associated with elevated D-dimer (1.37 [1.24–1.51] per 1 log_2_ mg/dL, p<.0001). In contrast, higher total (0.97 [0.96–0.98] per 10 mg/dL, p<.0001) and HDL cholesterol (0.98 [0.96–1.00] per 10 mg/dL, p<.0151) were found to be associated with lower D-dimer levels.

### Sensitivity Analyses

Logistic regression models yielded results highly consistent with linear models (data not shown). The factors associated with D-dimer levels did not differ between study participants off and on ART and between those with and without virological suppression (data not shown).

## Discussion

A better understanding of predictors of plasma D-dimer levels became particularly relevant in the light of new evidence indicating that both HIV+ [4],[5] and HIV- individuals [18], [19] with higher D-dimer levels are at a significantly increased risk of death. To our knowledge, this is the largest study investigating determinants of D-dimer published thus far. We have found that while a significant increase in D-dimer with age occurs in HIV+ men, HIV+ women have high D-dimer levels from an early age. These findings cannot be explained by an increased burden of co-morbidities or enhanced inflammation, as previously hypothesized. In those co-infected with HBV/HCV, hepatic fibrosis, but not hepatitis virus load, is independently associated with higher D-dimer. We also observed that HIV-specific variables, other demographic factors, biomarkers of inflammation, renal function and cholesterol levels are independently associated with higher D-dimer levels.

We found that black race, female sex and older age were demographic factors independently associated with higher D-dimer levels. African-American ethnicity was also found to be associated with higher plasma levels of D-dimer in HIV+ participants in the Veterans Aging Cohort Study (VACS) [10] and HIV- hypertensive adults [20]. Not surprisingly, the inter-racial variability in circulating D-dimer levels was shown to be, in part, genetically determined [21].

Increases in D-dimer levels with age have been previously reported [9], [10], [20], [22] and deleterious interactions between vascular damage, co-morbidities, inactivity and activated inflammation have been postulated as possible mechanisms [11], [12]. However, we did not find, except for hepatitis, significant associations between co-morbidities and D-dimer levels. Moreover, we found no significant interaction between age and inflammatory biomarkers. Taken together, our findings suggest that the increase of D-dimer with age is primarily attributable to causes other than a higher burden of concomitant diseases or an age-related pro-inflammatory state. Since elevated D-dimer was found to be correlated with arterial disease severity [23], worsening subclinical atherosclerosis may play an important role.

We found a significant interaction between age and gender and demonstrated that older age was more strongly associated with higher D-dimer in men than in women. We hypothesize that the significantly higher D-dimer levels observed in younger women may be due to higher estrogen levels and higher immune activation. Pregnancy, hormone replacement therapy and estrogen-containing contraceptive pills increase plasma levels of procoagulant factors and are well-known risk factors for thromboembolism [24], [25], which suggests a potential interplay between estrogen and D-dimer. After estrogen levels fall (among post-menopausal women), the gender difference is then attenuated as determinants of D-dimer levels for both genders may be more related to similar clinical and environmental factors. HIV+ women have also been found to have higher activation of CD8^+^ T cells than HIV+ men with comparable HIV plasma levels [26] and this exacerbated immune activation may have contributed to the higher D-dimer levels observed primarily in pre-menopausal women.

Significantly higher D-dimer levels have been seen in HIV-infected patients with ongoing viral replication and lower CD4^+^ cell counts [4], [6], [10]. Individuals receiving ART had significantly lower D-dimer levels than those off ART at baseline, but no remarkable differences between PI- and NNRTI-based regimens were noted. The factors independently associated with elevated D-dimer levels, however, did not differ considerably between individuals with suppressed or unsuppressed plasma HIV RNA levels. The control of HIV viral replication, therefore, did not substantially affect the main factors driving coagulation, a finding that suggests the potential benefit of adjunctive anti-thrombotic therapies during HIV infection, even in those with HIV viral suppression.

The positive correlation between nadir CD4^+^ cell counts and D-dimer levels that we observed was surprising and counter-intuitive. Nadir CD4^+^ counts were significantly associated with D-dimer levels only after adjustment for baseline CD4^+^ counts, but not in univariable analysis. The interplay between D-dimer levels, CD4^+^ cell counts and plasma HIV RNA is complex with dynamic changes after ART initiation [1], [5], [6] and this may in part be explained by lower D-dimer levels in participants who had good response to ART and presented a large increase from nadir to baseline CD4^+^ counts. However, this observation may have been a chance finding and further investigation is required.

Congruent with previous reports [4], [21], D-dimer and biomarkers of inflammation were positively correlated. Inflammatory responses promote fibrin formation and lysis, resulting in elevated D-dimer levels [27], with IL-6 being shown to directly activate the coagulation cascade [28]. Furthermore, D-dimer and other fibrin degradation products have been found to modulate the production of IL-6 and other inflammatory mediators [29]. This is consistent with a bi-directional interplay between inflammation and coagulation. We have also confirmed a previously reported negative correlation between HDLc and D-dimer [30]. Indeed, HDLc has been shown to down-regulate thrombotic pathways by multiple mechanisms, including inhibition of endothelial and platelet activation, promotion of endothelium-dependent vasodilatation and attenuation of thrombin generation [31].

We demonstrated for the first time that the degree of hepatic fibrosis, as demonstrated by higher hyaluronic acid levels, but not the replication of hepatitis viruses, was associated with higher D-dimer levels in co-infected patients. This is consistent with data from cirrhotic, HIV-uninfected individuals, in whom D-dimer levels were found to increase as hepatic impairment progresses [32]. However, similarly to other fibrosis markers, HA is not liver specific and may reflect other pathologies. Therefore, information on other measures of hepatic fibrosis, such as Fibroscan, APRI and FIB-4, would have been helpful. Of interest, recent data demonstrated that HIV replication, in part through associated reductions in levels of hepatocyte-dependent anti-coagulant factors, leads to a net-procoagulant state [33]. Additional research is needed to better understand the potential consequences of hepatic function for coagulation abnormalities, and clinical risk, among HIV positive patients.

Decreased renal function, as demonstrated by lower eGFR, was associated with elevated D-dimer in partially adjusted models in this study. We observed, however, that this association became no longer significant after adjustment for cystatin C, which, in turn, was found to be positively correlated with D-dimer levels. This finding indicates that cystatin C, as a surrogate measure of renal impairment, is a better predictor of D-dimer levels than eGFR. Higher D-dimer levels in renal failure may reflect both decreased D-dimer clearance and increased fibrin turnover [34], as well as an inherent pro-inflammatory state [35].

A number of caveats need to be noted regarding the present study. First, its cross-sectional design hampered our ability to infer causality and to characterize associations over time. Second, as data on variables of interest were not uniformly collected in the three trials, adjustment for important co-variates had to be done in smaller datasets. Conversely, given the large sample size of this study, some of the statistically significant associations we have found may not be clinically relevant. Finally, we have not investigated factors found to be associated with higher D-dimer levels in the general population, such as blood pressure, alcohol intake and physical activity [36].

In conclusion, D-dimer levels increase with age in HIV+ men, but are already high in women at an early age. This seems to be primarily attributable to causes other than a higher burden of concomitant diseases or an age-related pro-inflammatory state. In those with HBV/HCV co-infection, the only co-morbidity found to be associated with raised D-dimer, hepatic fibrosis, but not hepatitis virus replication, seems to influence D-dimer levels. The control of viral replication did not substantially affect the main factors driving coagulation in HIV+ persons and the role of adjunctive anti-thrombotic therapies should be investigated in this population. As only 20% of D-dimer variance could be explained by the factors we investigated, further studies on genetic, socio-economic and clinical correlates of D-dimer in HIV+ individuals are warranted. Prospective studies and randomized trials are also needed to determine whether pharmacologic interventions to lower elevated D-dimer levels can reduce morbidity and all-cause mortality during HIV infection. We believe that our findings can be instrumental in building the basic knowledge and in selecting suitable candidates for such studies.
